# The human kidney capsule contains a functionally distinct mesenchymal stromal cell population

**DOI:** 10.1371/journal.pone.0187118

**Published:** 2017-12-05

**Authors:** Daniëlle G. Leuning, Marten A. Engelse, Ellen Lievers, Roel Bijkerk, Marlies E. J. Reinders, Hetty C. de Boer, Cees van Kooten, Ton J. Rabelink

**Affiliations:** Department of Internal Medicine, division of Nephrology, Einthoven laboratory of Vascular and Regenerative Medicine; Leiden University Medical Centre, Leiden, The Netherlands; Center for Molecular Biotechnology, ITALY

## Abstract

We recently demonstrated that the adult human kidney cortex contains a perivascular stromal cell (kPSC) that shows organotypic properties and is important for repair and stabilisation of kidney function. Not only the kidney cortex but also the kidney capsule contains stromal cells that are important for the three dimensional organisation of the kidney during nephrogenesis. They provide the barrier function of the capsule which is critical for homeostatic processes such as pressure natriuresis. We postulated that stromal cells derived from the kidney capsule may therefore also have specific properties and functions. To this end, we isolated these capsule mesenchymal stromal cells (cMSC) from human cadaveric kidneys that were not suitable for transplantation. There were several similarities between cMSCs and kPSCs including support of vascular plexus formation, phenotypic marker expression and resistance against myofibroblast transformation. However, compared to kPSCs, cMSCs showed distinct mRNA and miRNA expression profiles, showed increased immunosuppressive capacity, and displayed strongly reduced HGF production, contributing to the inability to enhance kidney epithelial repair. Therefore cMSCs are a distinct, novel human kidney-derived MSC-population and these data underpin the large functional diversity of phenotypic similar stromal cells in relation to their anatomic site, even within one organ.

## Introduction

The kidney capsule consists of a layer of stromal cells surrounded by a sheet of connective tissue and is of major importance during kidney development and adult kidney homeostasis. During kidney development 3 different locations of stromal cells can be observed; capsular, cortical and medullary stromal cells. Nephrogenesis takes place in the outer nephrogenic zone, the region just below the kidney capsule [1–4]. Stromal cells within the kidney capsule are of major importance for nephrogenesis. Normally the kidney capsule consists of a continuous layer of Foxd1 and Hox10 positive stromal cells. However, in Foxd1-null embryos, the capsule contains several different cell types including endothelial cells and Bmp4- expressing cells resulting in delayed and disorganized nephrogenesis. Moreover, the defects in capsule formation resulted in adherence to the body wall and failure of kidney ascension which leads to fused kidneys on a pelvic location [5]. Comparable to Foxd1-null mice, triple mutants of the renal stromal marker Hox10 also show a failure of capsule formation with similar effects on nephrogenesis [6].

In the adult kidneys, the kidney capsule is important for kidney homeostasis. The strong layer of connective tissue is of importance for preserving the renal interstitial hydrostatic pressure (RHIP) which is necessary for pressure natriuresis. Decapsulation of the kidney in rats resulted in lower RIHP and reduced pressure-natriuretic response [7]. Moreover, in adult mice, stromal cells from the kidney capsule could be isolated and showed mesenchymal stromal cell (MSC) features such as the ability to adhere to plastic, the presence of MSC-markers such as Sca-1, trilineage differentiation capacity and clonogenicity. Moreover, these murine capsule MSCs were able to migrate into the injured kidney after ischemic injury and seemed to protect against kidney injury as decapsulation of the kidneys resulted in deceleration of recovery of kidney function [8]. However, little is known about the human kidney capsule.

We recently isolated and characterised perivascular stromal cells from the human adult kidney cortex (kPSC) and compared them to mesenchymal stromal cells (MSC) from the bone marrow (bmMSC) [9]. bmMSCs are immunomodulatory cells with reparative properties and have shown beneficial effects in kidney diseases and transplantation [10–12]. We showed that, although there are similarities between bmMSCs and kPSCs, there are also major differences. kPSCs show a different, organotypic gene expression, most notably the renal homeobox genes, Hox10 and Hox11, as well as different function. For example, in contrast to bmMSCs, they had the capacity to reintegrate into the renal cortex and stimulated kidney tubular epithelial repair [9]. These data suggest that kPSCs may have retained organotypic properties. In this respect, we were interested to see whether stromal cells isolated from the human renal capsule would show distinct functionalities compared to the cortical kPSCs based on their site-specific role during organogenesis.

Here we show that within the human kidney stromal cells of the kidney capsule share common general characteristics of MSCs, but yet are functionally distinct from the cortical kidney perivascular stromal cells (kPSCs) we previously described.

## Materials and methods

### Isolation and expansion of human kidney capsule-derived MSCs (cMSC)

Cells were isolated from kidney capsules from human transplant grade kidneys not suitable for transplantation due to surgical reasons. Specific research consent was given for all kidneys by either the donor, confirmed by the next of kin or by the next of kin directly according to Dutch legislation. None of the transplant donors were from a vulnerable population. The study was approved by the local medical ethical committee of the Leiden University Medical Centre (p13.054) and the ethical advisory board of the European Union consortium STELLAR. Kidney capsules were carefully removed from the kidney and pericapsular fat was removed. Slices of 1 cm^3^ were made and cultured with the kidney side down in a small layer of alphaMEM (Lonza, Verviers, Belgium) containing 5% platelet lysates (PL), 1 mmol/L glutamine (Lonza) and penicillin/streptomyozine (Lonza) in a T75 cell culture flask until outgrowth culture was obtained. Cultures were maintained at 37°C and 5% carbon dioxide. Half of the medium was refreshed twice a week. After confluency was reached, cells were collected using trypsin (Lonza) and replated at 4x10^3^ cells/cm^2^. Experiments were performed with cMSCs between passage 4–8. The population doubling was calculated with the following formula: Cumulative PD = Σlog(N1/N0). PD is population doubling, N1 the accumulated cell number at the end of incubation and N0 the accumulated cell number at the beginning of incubation.

### Isolation and expansion of human kidney cortex perivascular stromal cells (kPSC)

kPSCs were isolated from human transplant grade kidneys as described previously [9]. Shortly, after removal of the kidney capsule for cMSC isolation, kidneys were perfused via the renal artery with collagenase (2500 units, NB1, Serva) and DNAse (2,5ml Pulmozyme, Genetech) at 37°C with a flow of 100ml/min. After approximately 30 minutes, the tissue was digested and the resulting cell suspension was washed and collected. Cells were either directly cultured or frozen in liquid nitrogen. Kidney cell suspensions were cultured in alphaMEM (Lonza) containing 5% platelet lysates, glutamine (Lonza) and penicillin/streptomyzine (lonza). At passage 1 NG2 cell enrichment was performed using MACS according to manufacturer’s protocol (Miltenyi Biotech, Gladbach, Germany) and afterwards cells were cultured in alphaMEM containing 5% platelet lysates. Experiments were performed between passage 4–8. bmMSCs were isolated as described previously [9].

### Morphology and immunophenotype analysis

Cells were imaged with an inverted bright-field microscope (Leica DFC 295). For immunophenotyping, the cells were directly labelled for NG2, PDGFR-β, CD146, CD73, CD90, CD31, CD34, CD45, CD56, HLA class I (ABC),HLA class II (DR) (BD Bioscience, Franklink Lakes, NJ, USA) and CD105 (Ancell Corporation, Bayport, MN, USA) and analysed by flow cytometry (Accuri C6, BD Bioscience).

### Trilineage differentiation potential

cMSCs and kPSCs were cultured in adipogenic, osteogenic and chondrogenic medium according to manufacturer’s protocol (Lonza). Calcium deposits were shown with Alizarin Red in the osteogenic differentiation assay. In the adipogenic differentiation assay, lipid droplets were stained using Oil Red O. In the chondrogenic differentiation assay, cell pellets were formalin-fixed (4% PFA o/n) and embedded in paraffin. 5 μm sections were de-parafinnized, rehydrated and incubated with 1% toluidine blue for 20 minutes. All differentiations were analysed with an inverted bright-field microscope (Leica DFC 295).

### Microarray sample preparation and data analysis

RNA was isolated from biological triplicates of both cMSCs and kPSCs of the same donor using Trizol reagent (Life Technologies, Bleiswijk, the Netherlands) and the RNeasy kit (Qiagen, Heidelberg, Germany) according to the manufacturer’s protocols and as described previously [9]. RNA quality and quantity were assessed using the Bioanalyzer (Agilent Technologies, Santa Clara USA) and Nanodrop spectrophotometer (Nanodrop Technologies, Wesington, USA). Gene expression profiling was performed by Aros Applied Biotechnology (Aarhus, Denmark). cDNA and cRNA synthesis, labelling and hybridization on the Human HT12 V4 Gene Expression Beadchips (Illumina Inc., San Diego, USA) were performed according to manufacturer’s instructions. The beadchips were scanned using iSCAN system (Illumina Inc., San Diego, USA) and fluorescence intensities were uploaded into GenomeStudio Software (Illumina Inc., San Diego, USA). Genes with a detection p-value of >0.05 for all samples were excluded. Subsequent data was quantile normalized and the Pearson’s correlation coefficient was calculated (r^2^). Average signals>200 in either the kPSCs or cMSCs were considered above background levels. Differential expression was analysed using the gene expression module of Genome Studio (Illumina). For the table of the top differential expressed genes, genes were sorted on highest DiffScore (Illumina). For the heatmap the delta average signal of cMSCs vs. kPSCs was set to 200 (which is the threshold of the measurement) and the resulting 3034 genes selected were analysed for clustering in R software.

### miRNA profiling

For miRNA-profiling, reverse transcription of total RNA of cMSCs and kPSCs of 3 donors was performed using the miRNA reverse transcription kit (Thermo Biosystems, Foster City, CA). cDNA was preamplified using Megaplex PreAmp primers pools A V2.1 (Thermo Biosystems) according to manufacturer’s protocol. 384 miRNAs including six controls (RNU44, RNU48, 4*U6) were profiled using TaqMan^®^ Array MicroRNA Human Card A V2.0 (Thermo Biosystems). Samples were normalized to RNU48 expression. The heatmap of 2^40-CT^ values are shown for the top 250 differentially expressed microRNAs.

### TGF-β stimulation assay

cMSCs and kPSCs of three different donors were seeded in a density of 200.000 cells per well in a 6 wells plate (costar) and stimulated for 48 hours with 10 ng/ml TGF-β1 (PreproTech, London, United Kingdom). Afterwards, cells were trypsinized, permeabilized with 0.1% saponin and labelled with α-SMA (BD Bioscience). α-SMA expression was analysed with flow cytometry (Accuri C6, BD Bioscience) and mean fluorescent intensities were calculated (Kaluza, Beckman Coulter, Indianapolis, USA).

### Vascular plexus assay

Primary human glomerular-derived micro vascular endothelial cells (hgMVEC) were purchased from Cell Systems (ACBRI-128, Kirkland, WA) and cultured in EGM2 medium (Lonza). kPSCs or cMSCs of three different donors were cocultured with hgMVECs in a 96-wells plate (Costar, Sigma-Aldrich) for 1 week in a 4:1 ratio (hgMVEC:cMSC/kPSC) as described previously [13]. After 1 week, cells were fixated with ice-cold methanol (100%) for 10 minutes and endothelial sprouting was visualized with CD31 immune fluorescence (BD Bioscience, Franklin Lakes, NJ, USA, Zeiss LSM500). The percentage capillary coverage was analysed with imageJ software.

### Peripheral blood mononuclear cell isolation, proliferation assay and cytokine analysis

Peripheral blood mononuclear cells (PBMCs) were isolated from buffy coats of healthy blood donors obtained from the local blood bank by density gradient centrifugation using Ficoll-isopaque and frozen in liquid nitrogen until use. cMSCs of 3 donors and kPSCs of 2 donors were plated in triplo in flat-bottom 96-well plates (Costar, Sigma-Aldrich) and allowed to attach overnight in DMEM-F12 with 10% normal human serum (NHS) as described previously [9]. cMSCs and kPSCs were seeded in increasing cell numbers to eventually get a ratio of 1:128 (MSC:PBMC) (766 cells) to 1:4 (25.000 cells). PBMCs were stimulated with anti-CD3/antiCD28 Dynabeads (Invitrogen) and added to the kPSCs/cMSCs in a concentration of 1x10^5^ cells/well. After 5 days the supernatant was collected and ^3^H-thymidine (0.5 mCi) was added. After 16 hours ^3^H-thymidine incorporation was determined as a measure of proliferation. Cytokine excretion was analysed in the supernatant with the Bio-Plex Human Cytokine 17-Plex assay following manufacturer’s protocols (Bio-Rad Laboratories, Veenendaal, the Netherlands).

### Kidney epithelial scratch assay

cMSCs and kPSCs of 3 different donors were plated in a density of 200.000 cells/well in a 6 well culture plate (Costar, Sigma-Aldrich) and cultured for 48 hours [9]. Subsequently, the supernatant was collected for the use as conditioned medium. Growth factors in the conditioned medium of cMSCs and kPSCs were measured using a custom made growth factor multiplex-elisa panel following manufacturer’s instruction. (R&D systems, Minneapolis, USA). Proximal tubular epithelial cells (PTEC), HK-2 [14] were seeded in PTEC medium consisting of a Dulbecco’s modified Eagle’s medium (DMEM) F-12 (Lonza) supplemented with insulin (5μg/ml), transferrin (5μg/ml), selenium (5ng/ml), hydrocortisone (36 ng/ml), tr-iodothyrinine (40 pg/ml) and epidermal growth factor (10 ng/ml) (Sigma-Aldrich) in a density of 500.000 cells/well in a 6 wells cell culture plate (Costar) and cultured until confluent. A scratch wound was created in the monolayer of HK-2 cells using a 200μl pipette tip. After the scratch, cells were washed with PBS and provided with either fresh medium (alphaMEM+5%PL) or with complete conditioned medium from either cMSCs or kPSCs. Scratches were imaged at 3, 6, 12 and 24 hours at the same position in duplicates with an inverted bright-field microscope (Leica DFC 295). The scratch area was measured at each time point using ImageJ software and the percentage wound closure was calculated.

### Statistical analysis

Statistical analysis was performed with Graph Pad Prism (Graph Pad Prism Software Incl. San Diego, USA). Differences between kPSCs and cMSCs were analysed using either an unpaired two-tailed t-test or, when more variables are analysed a two way ANOVA with Bonferroni’s posthoc comparison analysis. Differences were considered statistically significant when p<0.05.

## Results

### MSC-like cells can be isolated from the human kidney capsule

**Fig 1:**
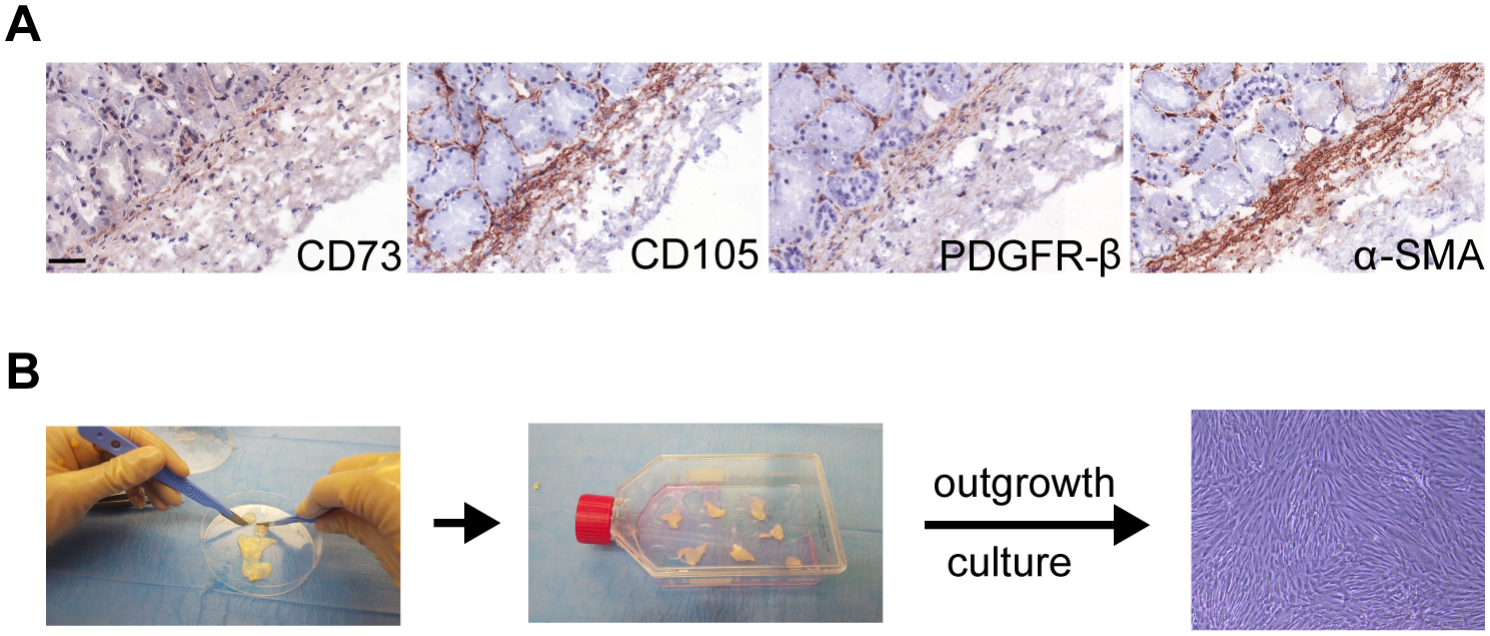
cMSC localization and isolation method. A) The human kidney capsule contains a layer of cells which is positive for the stromal markers CD73, CD105, PDGFRβ and α-SMA. B) Outgrowth culture of the human kidney capsule gives rise to spindle shaped cells. Abbreviations: PDGFR-β: platelet-derived growth factor receptor beta, α-SMA: alpha smooth muscle actin. Scale bar 50μm.

**Fig 2:**
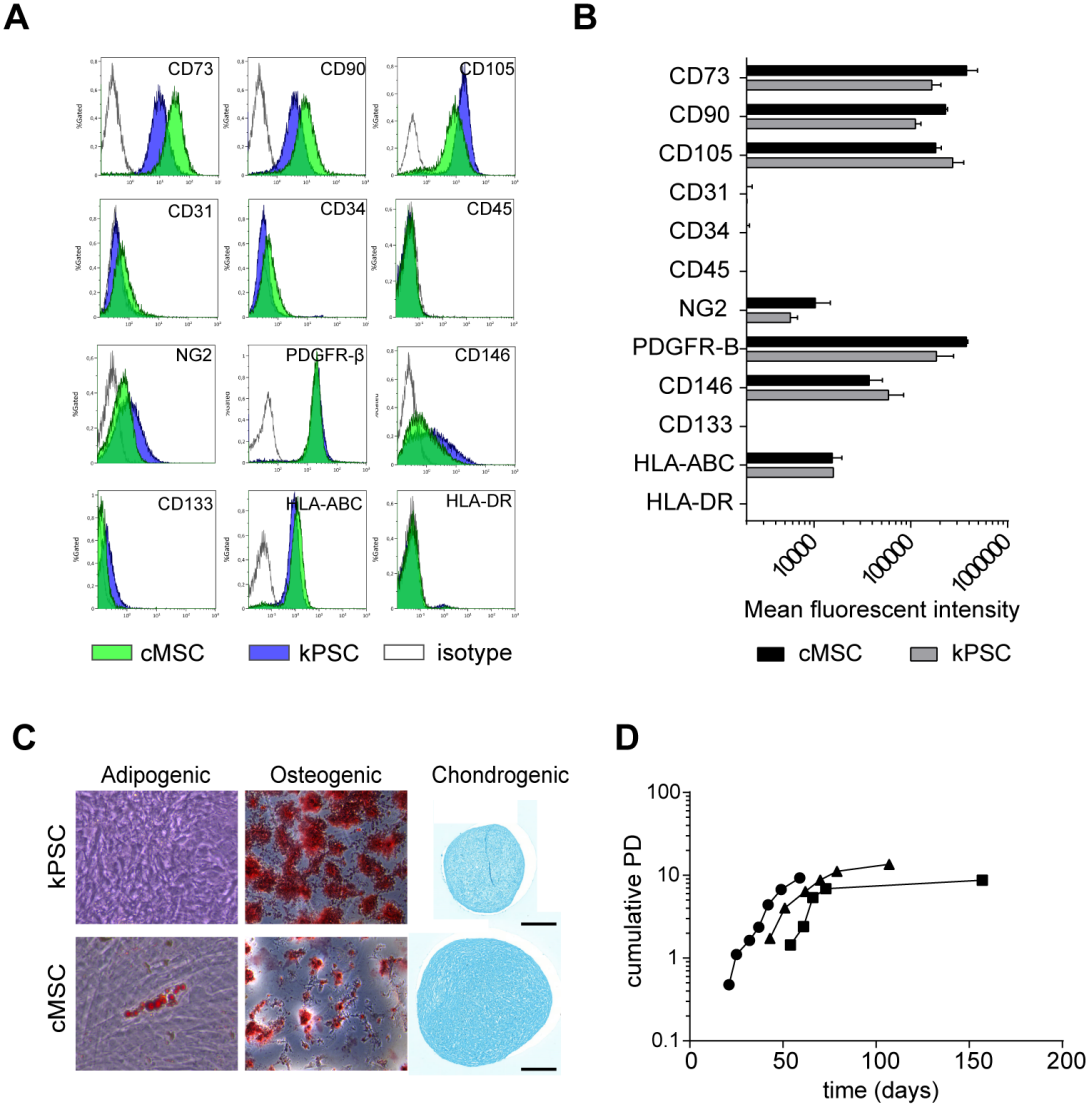
Characterization of cMSCs. A) cMSCs are positive for MSC markers CD73, CD90 and CD105 while being negative for CD31, CD34, and CD45. This marker expression was quantified by mean fluorescent intensities (MFI) of three different donors and compared to the MFIs of kPSCs of the same donors (B). C) cMSCs are able to differentiate into cartilage, bone and fat. The latter was not observed in kPSCs. D) Growth characteristics of cMSCs. Abbreviations: cMSC: human kidney capsule-derived mesenchymal stromal cells. kPSC: kidney cortex-derived perivascular stromal cells. PDGFR-β: platelet-derived growth factor receptor beta, HLA: human leucocyte antigen, PD: population doubling. Scale bar 250μm.

In order to analyse the presence and phenotype of stromal cells in the adult human kidney capsule, we stained the kidney capsule for the stromal markers CD73, CD105, PDGFR-β and α-SMA. All these markers are expressed in both the kidney capsule and in the perivascular regions of the cortex (Fig 1A). Via outgrowth culture of the kidney capsule on cell culture plastic, spindle shaped cells could be obtained (cMSC) (Fig 1B). Maker expression of cMSCs isolated from three donors were compared to the kidney perivascular stromal cells (kPSC) sorted from the same kidneys based on NG2-expression as we described previously [9]. cMSCs were, like kPSCs positive for the surface markers CD73, CD90, CD105 and HLA-ABC while being negative for CD31, CD34, CD45 and HLA-DR (Fig 2A). This marker expression was robust and similar to the marker expression of kPSCs isolated from the same donors (Fig 2B). cMSCs showed chondrogenic, osteogenic and adipogenic differentiation capacity, which is different from kPSCs which lack adipogenic differentiation (Fig 2C). In contrast to kPSCs, there was no NG2-enrichment step needed to isolate cMSCs. As shown in Fig 2D, there is, as observed with other MSC populations, donor variation in growth characteristics in both population doubling and in the moment of reaching senescence. Senescence was observed between 50–70 days of culture.

### cMSCs show a distinct mRNA and miRNA expression profile

**Fig 3:**
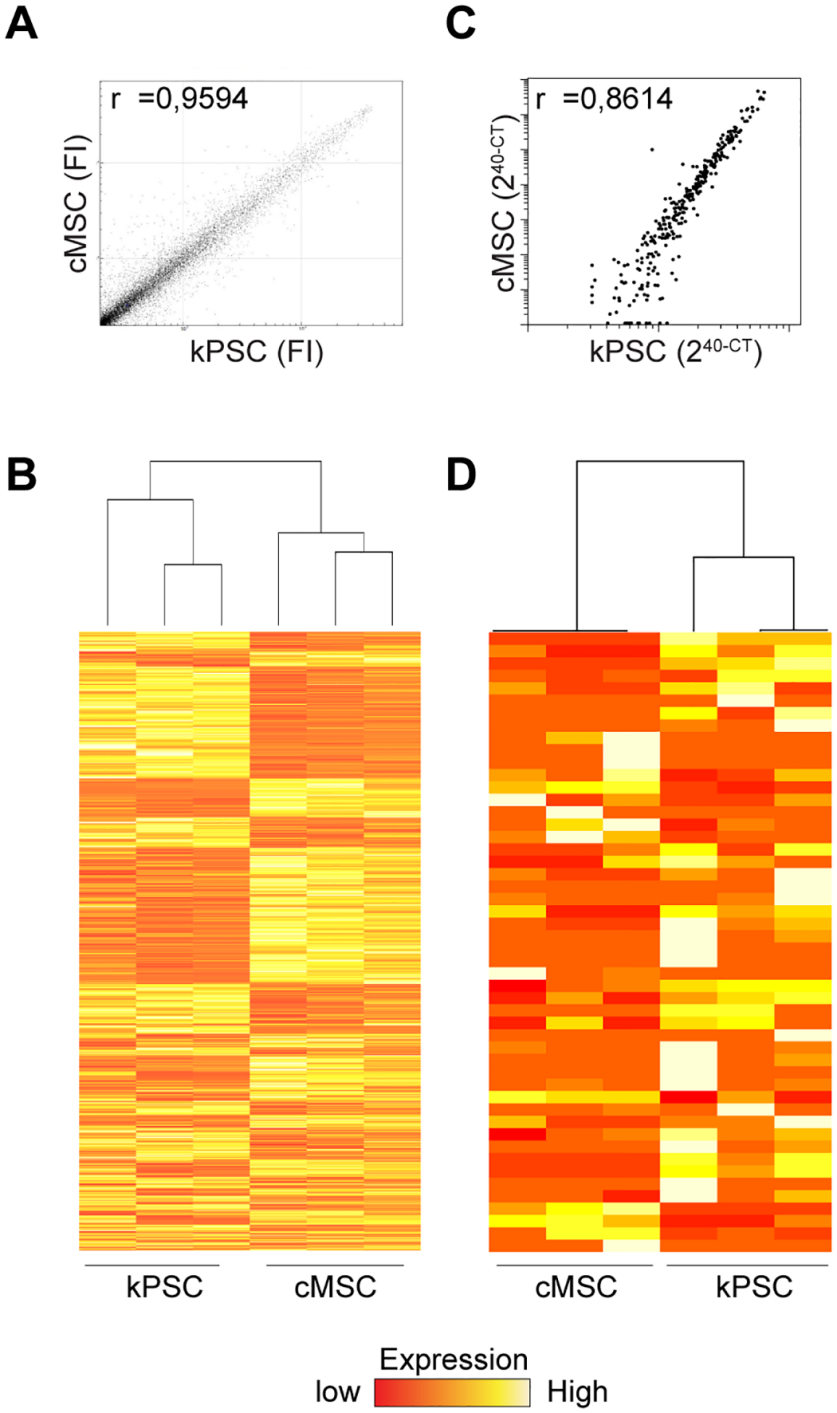
Transcriptome analysis of human cMSCs compared to kPSCs of the same donor kidneys. A) cMSCs and kPSCs show in general similar expression profiles when comparing all transcripts (35000) as depicted by a Pearson correlation of 0,9594. B) When analyzing the 3000 differentially expressed genes, cMSCs and kPSCs show hierarchical clustering according to isolation area and not according to donor as depicted in the dendrogram and heatmap. C) When comparing all 315 analyzed miRNAs, cMSCs and kPSCs showed a similar miRNA expression profile (Pearson correlation coefficient of 0.86) with hierarchical clustering into cMSC and kPSCs as depicted in the dendrogram and heatmap of the top 50 differentially expressed miRNAs. (D). Biological triplicates. Abbreviations: cMSC: kidney capsule-derived MSC, kPSC: kidney cortex perivascular stromal cell, FI: fluorescent intensity. GEO accession number GSE101973, https://www.ncbi.nlm.nih.gov/geo/query/acc.cgi?acc=GSE101973.

In order to evaluate the differences between cMSCs and kPSCs in more detail, Illumina microarray expression profiling was performed on biological triplicates. kPSCs and cMSCs were derived from the same donors. 35000 transcripts were analysed and cMSCs and kPSCs had a similar expression profile of most genes with a Pearson correlation coefficient of 0,9594 (Fig 3A), suggesting high similarities between the cell types. However, still approximately 3000 genes were differentially expressed. Hierarchical clustering showed clustering between cMSCs and kPSCs indicating more similarities between cMSCs of different donors, than between cMSCs and kPSCs of the same donor (Fig 3B). This suggests that cMSC and kPSC are distinct cell populations. Table 1 shows the top 5 up and down regulated genes comparing the two cell types. The top 50 differentially expressed genes can be found in the supporting information (S1 Table) Expression profiles of cMSCs were also compared to those of bmMSCs and also here cMSCs and bmMSCs clustered according to cell source (S1A Fig). Similar results were observed for the expression profile of miRNAs. Most miRNAs showed a similar expression profile with a Pearson correlation coefficient of 0.8614. However, 114 out of 315 miRNAs showed a fold increase of 2 or more (S2 Table) and again hierarchical clustering was observed according to cell source and not according to donor as depicted in the heatmap (Fig 3D) The heatmap of cMSCs compared to bmMSCs can be found in the supporting information (S1B Fig).

**Table 1:** Top 5 differentially expressed genes.

**Top 5 enriched genes cMSC**					
Gene name	Symbol	Average signal cMSC (SD)	Average signal kPSC (SD)	FI	DiffScore
microfibrillar-associated protein 4	MFAP4	6273 (943)	636 (318)	9,9	344,6
protein S (alpha)	PROS1	2120 (358)	354 (70)	6,0	344,6
fibronectin leucine rich transmembrane protein 2	FLRT2	2293 (153)	394 (52)	5,8	344,6
cellular retinoic acid binding protein 2	CRABP2	2122 (257)	428 (100)	5,0	344,6
secernin 1	SCRN1	2319 (70)	506 (168)	4,6	344,6
**Top 5 enriched genes kPSC**					
Gene name	Symbol	Average signal cMSC (SD)	Average signal kPSC (SD)	1/FI	DiffScore
coiled-coil domain containing 81	CCDC81	167 (7,5)	443 (17)	2,7	-258,0
endothelin 1	EDN1	498 (101)	2209 (242)	4,4	-255,7
nestin	NES	230 (29)	942 (103)	4,1	-246,0
translocation associated membrane protein 2	TRAM2	1377 (238)	3274 (187)	2,4	-217,2
keratin 34	KRT34	178 (9)	416 (20)	2,3	-196,0

Upper table: enriched genes in cMSC based on differential score (DiffScore). Lower table: enriched genes in kPSCs. Abbreviations: FI: fold increase, SD: standard deviation, cMSC: kidney capsule-derived MSC, kPSC: kidney cortex- derived perivascular stromal cell

### cMSCs did not transform into myofibroblasts after stimulation with TGF-β

**Fig 4:**
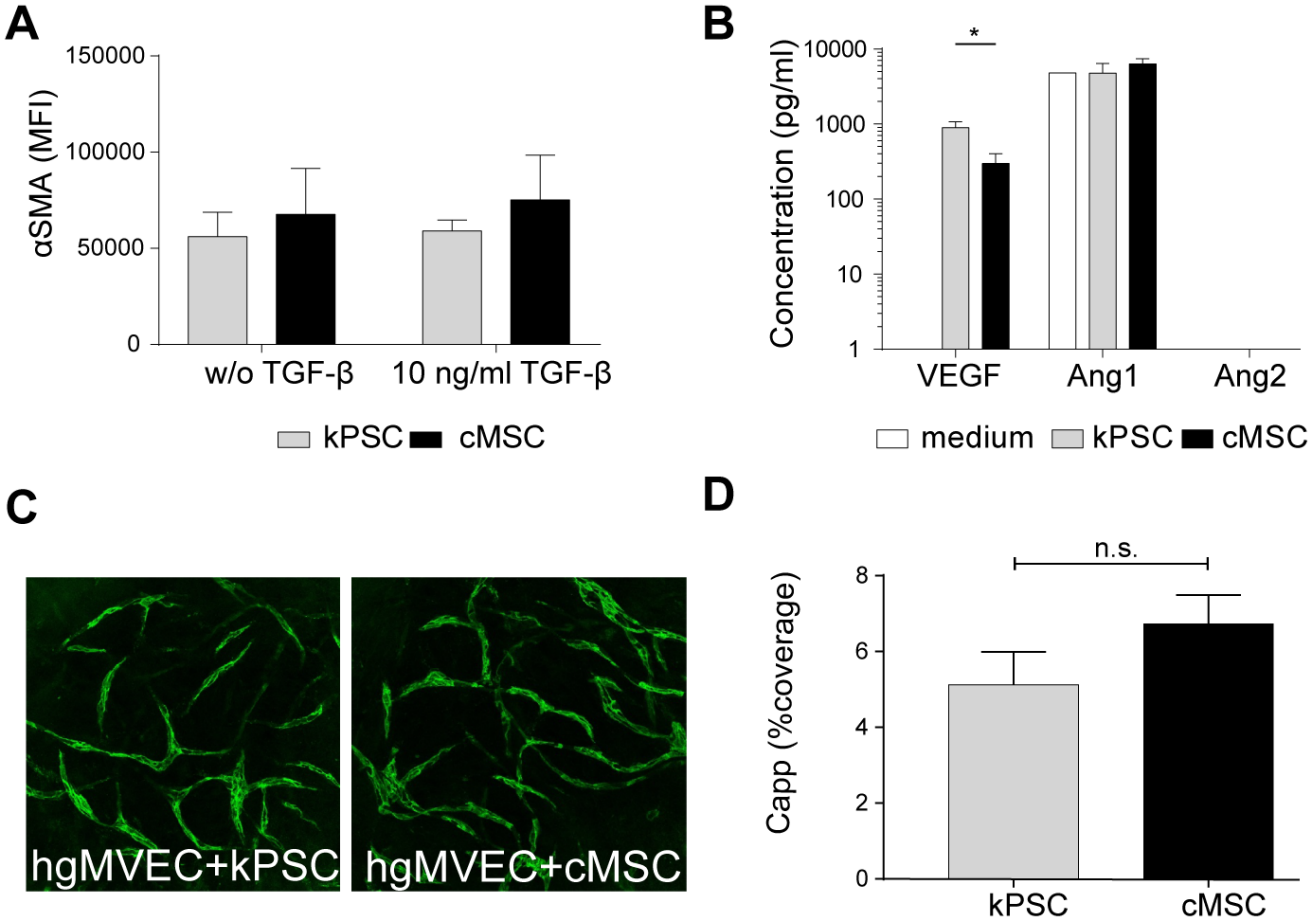
Functional similarities between cMSCs compared to kPSCs. A) Analysis of α-SMA expression in kPSCs and cMSCs after TGF-β stimulation show no up regulation of α-SMA. B) Excretion of angiogenic factors VEGF, Ang1 and Ang2 by kPSCs and cMSCs. cMSC excrete less VEGF compared to kPSCs. C) representative CD31 immunofluorescence of hgMVECs in coculture with either kPSCs or cMSCs show capillary network formation D) There is no significant difference in capillary coverage comparing kPSCs with cMSCs. Abbreviations: cMSC: human kidney capsule-derived mesenchymal stromal cell, kPSC: kidney cortex-derived perivascular stromal cell, α-SMA: alpha smooth muscle actin, TGF-β: transforming growth factor beta, w/o: without, MFI: mean fluorescent intensity. hgMVEC: human glomerular micro vascular endothelial cells, capp: capillary coverage, VEGF: vascular endothelial growth factor, Ang: angiopoietin, n.s.: non-significant. *p<0.05.

As the kidney capsule contains a strong fibrous layer we evaluated whether cMSCs have the capacity to transform into myofibroblasts. However, comparable to kPSCs, cMSCs did not show an increased a-SMA expression after stimulation with TGF-β (Fig 4A). This indicates that cMSCs do not transform into myofibroblasts upon *in vitro* stimulation. This is in contrast to bmMSCs for which we previously showed that they do increase α-SMA expression upon stimulation [9].

### Vascular network formation was stabilized by coculture of human glomerular endothelial cells with cMSCs and kPSCs

Due to their perivascular location, we hypothesized that kPSCs are able to stabilize endothelial cells to a greater extent than cMSCs. When looking at angiogenic growth factors, kPSCs indeed excrete vascular endothelial growth factor (VEGF) in higher levels than cMSCs. No differences were observed in angiopoietin 1 (ang1) excretion (Fig 4B). To assess the functional consequences, cMSCs and kPSCs were cocultured with human glomerular micro vascular endothelial cells (hgMVECs). hgMVECs alone were not able to form vascular networks, however, coculture with either cMSCs or kPSCs gave rise to the formation of vascular tubular structures in a vascular network (Fig 4C). There were no differences in the amount of vascular tubular structures as quantified in Fig 4D.

### cMSCs are more immunosuppressive compared to kPSCs

**Fig 5:**
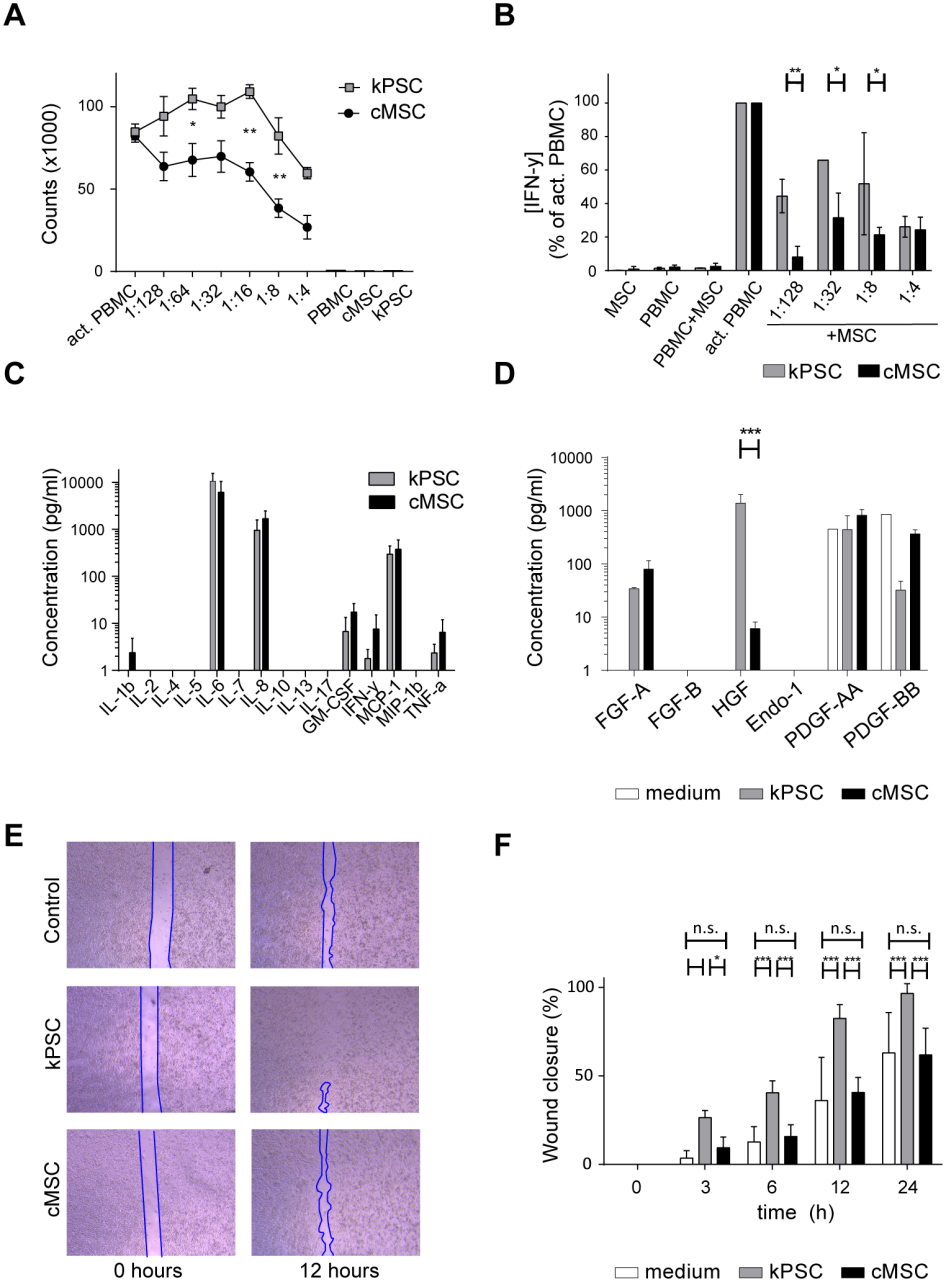
Functional differences between cMSCs and kPSCs. A) Within a PBMC suppression assay, cMSCs were able to suppress PBMC proliferation significantly more than kPSCs. B) cMSCs were able to decrease the IFN-y excretion in the PBMC suppression assay, indicating more immunosuppression by cMSCs. C) Unstimulated kPSCs and cMSCs excreted similar levels of major cytokines. D) When looking at growth factors, cMSC-conditioned medium contained a 200-fold lower concentration of HGF compared to kPSCs while no significant differences were observed in other growth factors. E) While kPSCs are able to accelerate wound closure in a kidney epithelial wound scratch assay, cMSCs did not show this potential, as quantified in F). Abbreviations: cMSC: human kidney capsule-derived mesenchymal stromal cell, kPSC: kidney cortex derived perivascular stromal cell, act.: activated, PBMC: peripheral blood mononuclear cells, IFN-γ: interferon gamma, GM-CSF: granulocyte-macrophage colony-stimulating factor, MCP-1: monocyte chemo attractant protein-1, MIP-1b: macrophage inflammatory protein-1beta, TNF-a: tumor necrosis factor alpha, FGF: fibroblast growth factor, HGF: hepatic growth factor, Endo-1: endothelin 1, PDGF: platelet-derived growth factor. *p<0.05, ** p<0.01, ***p<0.001.

To evaluate the immunosuppressive capacity of cMSCs a peripheral blood mononuclear cell (PBMC) suppression assay was performed. In this assay, PBMCs were activated with polyclonal CD3/CD28 activation in the absence or presence of increasing amounts of cMSCs or kPSCs. While kPSCs were only able to inhibit PBMC proliferation at high MSC:PBMC ratios (1:8 and 1:4), cMSCs already showed an effect at lower cell numbers and showed significantly more inhibition than kPSCs (Fig 5A). This is to a similar extent compared to the inhibition by bmMSCs (S2A Fig). Moreover, the excretion of the pro-inflammatory cytokine IFN-y by the activated PBMCs was lower in coculture with cMSCs than in coculture with kPSCs indicating more immunosuppression by the cMSCs (Fig 5B). When looking at general cytokine excretion profiles, unstimulated cMSCs and kPSCs excrete similar levels of major cytokines (Fig 5C). However, the secretome of unstimulated MSCs does not reflect their functionality while the decrease in IFN-y excretion by PBMCs does.

### cMSCs were not able to enhance human kidney epithelial repair

We previously showed that the conditioned medium of kPSCs was, in contrast to the conditioned medium of bmMSCs, able to accelerate kidney tubular epithelial repair in a kidney epithelial wound scratch assay and that this effect was HGF depended [9]. When looking at major growth factors, most growth factors showed similar excretion profiles, with the exception of HGF. HGF was excreted in a more than 200 fold lower concentration by cMSCs compared to kPSCs. This also resulted in a decreased epithelial wound healing by the conditioned medium of cMSCs as shown in Fig 5E and quantified in Fig 5F. This lack of kidney epithelial wound scratch healing capacity is similar to what is observed with bmMSCs as shown in the supporting information (S2B Fig).

## Discussion

Previously it has been shown that from most organs MSC-like cells can be isolated. These organ-derived MSC like cells are mainly isolated from the perivascular compartment and exhibit tissue specific properties [15, 16]. From the human kidney cortex perivascular stromal cells could also be isolated and these cells showed kidney-specific regeneration and integration properties when compared to bmMSCs [9, 17].

Here we show that within the human kidney, not only in the cortical perivascular compartment of the kidney but also in the kidney capsule, a MSC-like cell population is present. Although there were similarities between the cell types in for example marker expression and vascular stabilisation properties, there were also major differences. Though isolated from the same donors, cMSCs showed a distinct mRNA and miRNA expression profile with clustering according to cell type and not to donor. Moreover, cMSCs showed increased immunosuppressive capacities while lacking the properties to accelerate kidney epithelial wound healing. Thus, cMSCs are a distinct kidney-derived MSC-like cell population.

Only few studies have been performed comparing MSC-like cells isolated from one tissue. Functional differences have been described between different isolation sides of adipose tissue [18, 19]. Naftali-Shani et al., for example, showed that MSCs isolated from the pericardial fat, epicardial fat and subcutaneous fat showed distinct cytokine excretion profiles, mRNA expression profiles and reparative properties. Interestingly and unexpectedly, cardiac dysfunction after myocardial infarction was worst after transplantation of MSCs from the epicardial fat compared to subcutaneous fat MSC transplantation and control [18].

MSCs are usually studied as candidate cells for cellular therapies. Although the in-organ differential functionality of MSCs are of interest to our understanding of the structural biology of the kidney, cMSCs are less likely to be used as candidate for clinical therapies. In clinical trials usually 2 cell infusions of 1–2 million cells per kilogram body weight are given [12]. To obtain such cells numbers, cMSCs should be isolated from several different donors for infusion into one patient, which makes the use of cMSCs less feasible compared to other MSC sources.

This is the first description of the functional characteristics of human kidney capsule-derived MSCs. Moreover, this is the first extensive functional comparison between kidney cortex derived and kidney capsule-derived MSC-like cells. The differences observed between the cells types in gene expression profile and functionality may reflect site-specific differentiation during organogenesis.

## Conclusion

cMSCs are a distinct, novel human kidney-derived MSC-population which underpins the large functional diversity of phenotypic similar stromal cells in relation to their anatomic site, even within one organ.

## Supporting information

S1 Fig Gene and miRNA expression profiles of cMSCs compared to bmMSCs and kPSCs. A) Heatmap of mRNA expression profiles of cMSCs, bmMSCs and kPSCs show hierarchical clustering according to cell source. B) Heatmap of miRNA expression profiles. Abbreviations: cMSC: human kidney capsule-derived mesenchymal stromal cell, kPSC: kidney cortex derived perivascular stromal cell.
(TIF)

S2 Fig A) Within a PBMC proliferation assay cMSCs were able to supress PBMC proliferation significantly more than kPSCs and to a similar extent as bmMSCs. B) Both bmMSCs and cMSCs were not able to accelerate epithelial wound healing while kPSCs did show this potential. Abbreviations: cMSC: human kidney capsule-derived mesenchymal stromal cell, kPSC: kidney cortex derived perivascular stromal cell, act.: activated, PBMC: peripheral blood mononuclear cells, n.s non significant, *p<0.05, ** p<0.01, ***p<0.001.
(TIF)

S1 Table Top 50 differentially expressed genes between kPSCs and cMSCs. Quantile normalized average signals are shown. Abbreviations: kPSC: kidney cortex perivascular stromal cell, cMSC: kidney capsule-derived mesenchymal stromal cell.
(XLSX)

S2 Table Differentially expressed miRNAs. RNU48 normalized 2^(40-CT) values are shown. Abbreviations: kPSC: kidney cortex perivascular stromal cell, cMSC: kidney capsule-derived mesenchymal stromal cell.
(XLSX)
