# Supplementary figures and images for: The human kidney capsule contains a functionally distinct mesenchymal stromal cell population
Source: PLoS One. 2017 Dec 5;12(12):e0187118. doi: 10.1371/journal.pone.0187118 (PMC5716605; doi:10.1371/journal.pone.0187118)

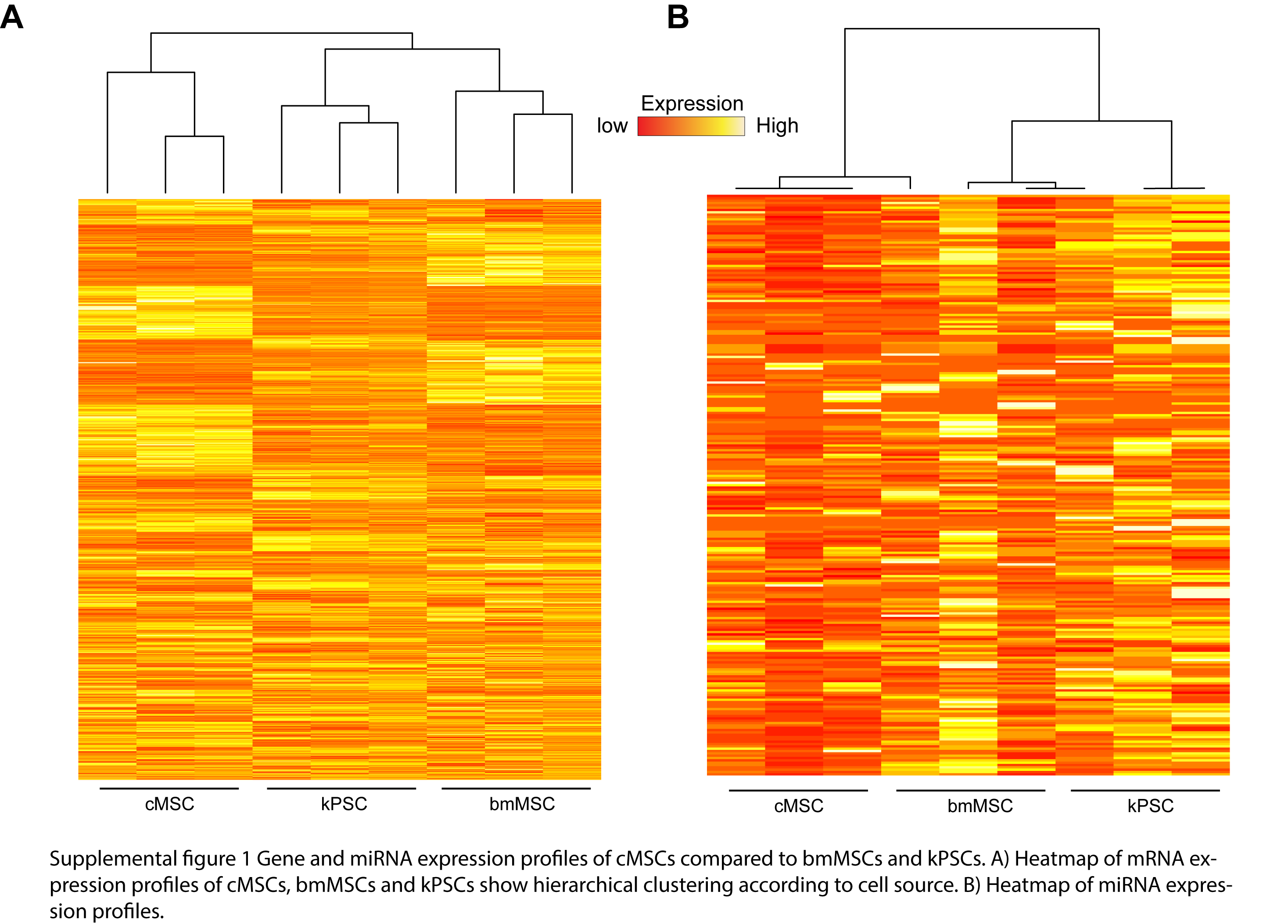

Supplement: S1 Fig — A) Heatmap of mRNA expression profiles of cMSCs, bmMSCs and kPSCs show hierarchical clustering according to cell source. B) Heatmap of miRNA expression profiles. Abbreviations: cMSC: human kidney capsule-derived mesenchymal stromal cell, kPSC: kidney cortex derived perivascular stromal cell. (TIF) [file pone.0187118.s001.tif]

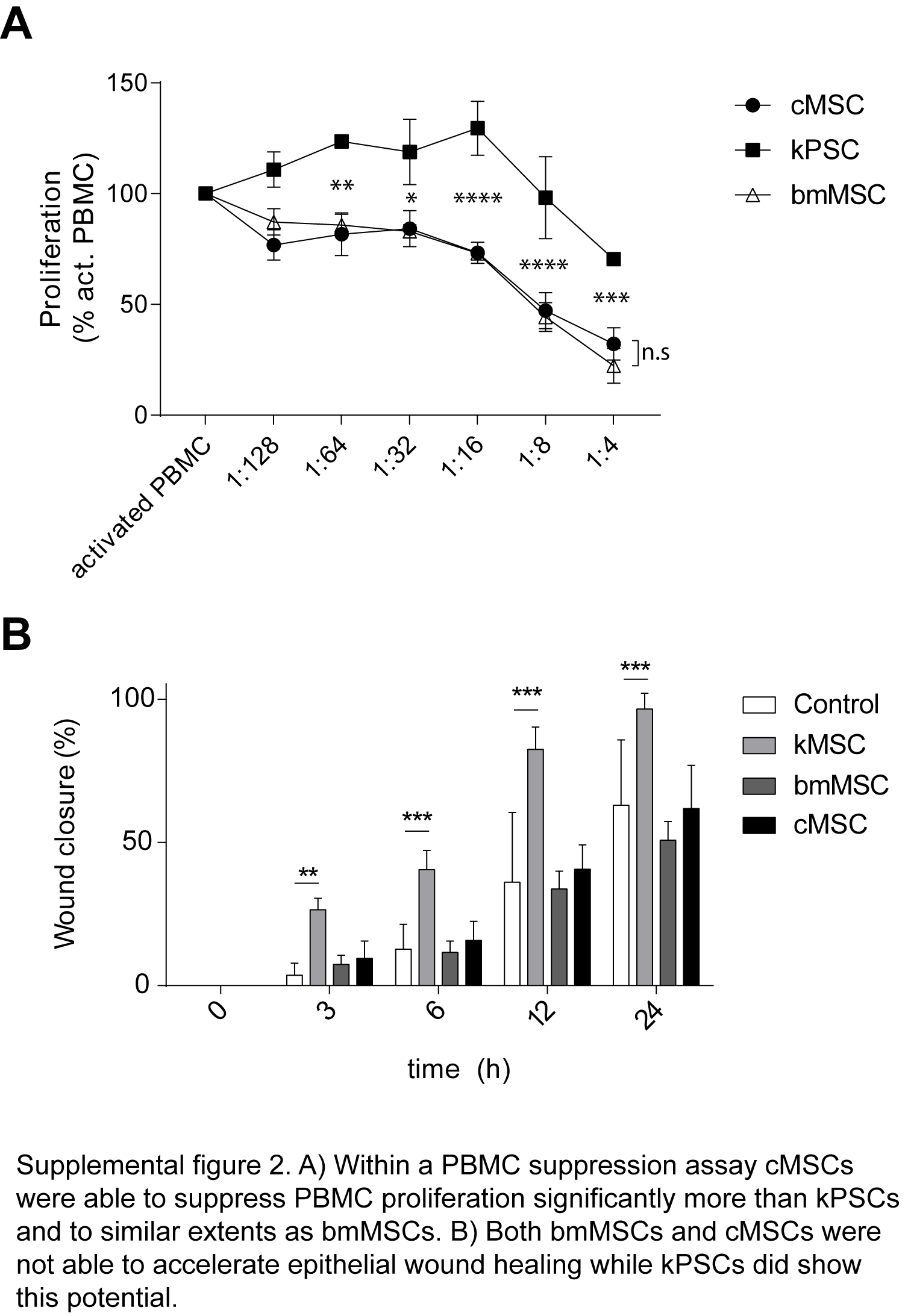

Supplement: S2 Fig — A) Within a PBMC proliferation assay cMSCs were able to supress PBMC proliferation significantly more than kPSCs and to a similar extent as bmMSCs. B) Both bmMSCs and cMSCs were not able to accelerate epithelial wound healing while kPSCs did show this potential. Abbreviations: cMSC: human kidney capsule-derived mesenchymal stromal cell, kPSC: kidney cortex derived perivascular stromal cell, act.: activated, PBMC: peripheral blood mononuclear cells, n.s non significant, *p<0.05, ** p<0.01, ***p<0.001. (TIF) [file pone.0187118.s002.tif]
